# Regional Analgesia in Intensive Care Unit

**DOI:** 10.5812/aapm.10587

**Published:** 2013-09-01

**Authors:** Mohammad Reza Hajiesmaeili, Mahsa Motavaf, Saeid Safari

**Affiliations:** 1 Department of Anesthesiology and Critical Care Medicine, Iran University of Medical Sciences (IUMS), Tehran, Iran; 2 Pain Research Center, Shahid Sadoughi University of Medical Sciences and Health Services, Yazd, Iran; 3 Rezvan Medical Research Center, Tehran, Iran

**Keywords:** Pain, Analgesia, Intensive Care Units, Epidural Anesthesia

Dear Editor, 

Acute pain has emerged as a leading stressor for ICU (Intensive Care Unit) patients, moderate to severe pain intensity which has been observed in nearly 50% of ICU patients. While pain is widely recognized as the so-called fifth vital sign and we believed pain relief is an essential human right (1, 2). Researchers demonstrated that 95% of physicians and 81% of nurses felt that the patient had adequate analgesia, whereas 74% of patients rated their pain as moderate or severe, in traumatic critically ill patients admitted to an ICU (3).

In traumatic and surgically critical ill patients, the pain associated with ventilatory function impairment and increases pulmonary morbidity such as nosocomial pneumonia, atelectasis and etc. (1) and in surgical ICU patients, early mobilization and rehabilitation with minimally associated pain and discomfort is the most desirable feature, reducing thromboembolic events an improved surgical outcome (4). Multiple modalities are available to the clinician for the treatment of pain in critically ill patients. A multimodal approach to pain management in the ICU patients should include the etiologic treatment of pain, intervention to reduce discomfort associated with positioning, fixation of invasive devices, physiotherapy for improved joint movement and prevention of muscle atrophy and pharmacological treatment (3). Uses of regional analgesic techniques allow maintenance of analgesia without the need for systemic and centrally acting agents and daily interruption of sedation.

Regional analgesia could provide single shout techniques for painful procedures in critically ill patients and also continuous techniques for prolonged analgesia. Richman demonstrated in a meta-analysis report that continuous peripheral nerve blocks provided superior postoperative pain relief and reduced opioid side effects (5). Single-shot and continuous peripheral nerve blocks in the critically ill include Interscalene block, Cervical paravertebral, Infraclavicular, Axillary, Paravertebral, Combination of femoral and sciatic block. In 2005, Schulz-Stübner et al., presented an summary of indications, limitations and practical aspects of regional anesthesia and analgesia in critically ill medical and surgical patients (3, 6). Regional analgesia using single-injection regional blocks and continuous neuraxial and peripheral catheters can play a valuable role in a multimodal approach to pain management in the critically ill patient to achieve optimum patient comfort and to reduce physiologic and psychological stress (3). Limitations for the use of regional anesthetic techniques are mainly associated with bleeding risks, hemodynamic side-effects, difficulties in neurologic assessment and the potential of local anesthetic toxicity (6). At present, continuous epidural analgesia remains currently the most commonly utilized regional analgesia technique in ICU patients (3) and epidural catheter could provide not only perioperative surgical anesthesia but also post-op analgesia in surgical ICU patients (7). Although, findings from meta-analysis of RCTs comparing epidural and intravenous PCA opioids are equivocal regarding analgesic efficacy (Category C1 evidence) (8). There are several studies that show benefits regarding outcome parameters like mortality, length of ICU stay, length of ventilator support, rate of nosocomial pneumonia, improving pulmonary function tests, incidence of ileus, improved analgesia or patient satisfaction (3, 9, 10). 

Immediate extubation after vast majority of patients, especially cardiac surgery and two-stage oesophagectomy in patients with thoracic epidural analgesia is safe and associated with low morbidity and mortality. This has cost-saving and logistical implications (11, 12). Indications for epidural catheters, in ICU patients, include; 1) Chest trauma and surgery, especially multiple rib fractures and esophageal surgery 2) Cardiac surgery 3) Abdominal surgery 4) Orthopedic surgery of pelvis and lower extremities 5) Others such as paralytic ileus, pancreatitis, cancer pain, intractable angina pain, pelvic malignancies, peripheral vascular disease of lower extremities (3). There are few contraindications to the placement of continuous epidural catheters. Absolute contraindications including patient refusal; a patient's inability to maintain stillness during the needle puncture, which can expose the neural structures to an unacceptable risk of injury; and raised intracranial pressure, which may predispose to brainstem herniation. Relative contraindications included Coagulopathy and anticoagulated patients, bacteremia or sepsis, local infections at the puncture site, severe hypovolemia, acute hemodynamic instability, obstructive ileus and lack of physician's experience. These conditions, especially use of anticoagulation for thromboembolic event, are common in the ICU, have led to an inevitable overlap between them and should be considered when selecting patients careful (13).

Odoom and Sih used continuous epidural analgesia (EA) without problem in 1000 vascular operations for patients receiving oral anticoagulants preoperatively (14). Despite lacking data, the use of EA in patients receiving prophylactic heparin is probably acceptable if the block can be performed, it is recommended that, the risk-benefit ratio must be determined for each patient (13). 

Epidural analgesia should be delayed for at least 10 to 12 hours after the last dose of LMWH, post procedure treatment with LMWH should be delayed at least 12 hours, and removal of the epidural catheters should take place 10 to 12 hours after the last dose, with subsequent dosing delayed for at least two hours (13). Epidural analgesia technique to the patients should be provided, including patient controlled EA, continuous EA, pre incisional EA, post incisional EA, cervical, thoracic and lumbar EA, using opioids, local anesthetics or sympatholytics such as dexmedthomidine. The new amide local anesthetic such as Ropivacaine has minimal hemodynamic effect, central nervous system toxicity and a lesser motor block during post-operative epidural analgesia (15). Opioids have been used traditionally as an adjunct for epidural analgesia in combination with local anesthetics. Opioid adjunct have a dose sparing effect of local anesthetic and better analgesia but there is always a possibility of an increased incidence of opioid side effects such as pruritis, urinary retention, nausea, vomiting and respiratory depression (16).

Dexmedetomidine is a class of alpha-2 agonist and It acts on both pre and post synaptic sympathetic nerve terminal and central nervous system, which has numerous beneficial effects including decreasing the sympathetic outflow and nor-epinephrine release causing sedative, anti-anxiety, analgesic, sympatholytic and hemodynamic effects (7, 17). Dexmedetomidine does cause a mild desirable hypotension and bradycardia but it is the lack of opioid side effects like respiratory depression, pruritis, nausea, and vomiting (17). 

By avoiding high systemic doses of opioids, several complications like withdrawal syndrome, delirium, mental status changes, and gastrointestinal dysfunction can be reduced or minimized. Because of limited patient cooperation during placement and monitoring of continuous regional analgesia, indications for their use must be carefully chosen based on anatomy, clinical features of pain, coagulation status, and logistic circumstances. High-quality nursing care and well-trained physicians are essential prerequisites to use these techniques safely in the critical care environment (3).
